# Development of Zinc-Doped Hydroxyapatite by Sol-Gel Method for Medical Applications

**DOI:** 10.3390/molecules23112986

**Published:** 2018-11-15

**Authors:** Catalin Constantin Negrila, Mihai Valentin Predoi, Simona Liliana Iconaru, Daniela Predoi

**Affiliations:** 1 National Institute of Materials Physics, Atomistilor Street, No. 405A, P.O. Box MG 07, 077125 Magurele, Romania; catalin.negrila@infim.ro (C.C.N.); simonaiconaru@gmail.com (S.L.I.); 2 Department of Mechanics, University Politehnica of Bucharest, BN 002, 313 Splaiul Independentei, Sector 6, 060042 Bucharest, Romania; predoi@gmail.com

**Keywords:** zinc-doped hydroxyapatite, sol-gel synthesis, ultrasound characterization

## Abstract

Zinc- (Zn) doped hydroxyapatite (HAp) were prepared by sol-gel method. Zinc-doped hydroxyapatite (ZnHAp) and HAp were analyzed by X-ray diffraction (XRD) and X-ray photoelectron spectroscopy (XPS). The Rietveld analysis revealed that the HAp and 7ZnHAp powders obtained by sol-gel method have a monophasic hydroxyapatite structure belonging to the P6_3/m_ spatial group. The results obtained from the ultrasound characterization of HAp and ZnHAp are also presented in this study. The effect of zinc concentration on properties that were deduced from ultrasonic measurements are studied in the case of a significant zinc concentration (x_Zn_ = 0.07). From the values of the ultrasonic waves velocities were determined by the pairs of elastic coefficients of the suspensions (Young modulus E, Poisson coefficient ν), which have proven to be similar to those determined by other authors.

## 1. Introduction

The most famous biocompatible materials generally used as coatings for implantable devices is hydroxyapatite (HAp). With the general chemical formula, Ca_10_(PO_4_)_6_(OH)_2_, HAp is a bioactive bioceramic belonging to the apatite family and is chemically similar to the mineral component of hard tissues found in mammals [1,2]. Therefore, due to its outstanding biological and physico-chemical properties, HAp, is widely used in biomedical applications for hard tissue replacements, scaffolds, as coating for implantable devices, and as a reinforcement material in biocomposites [3,4,5,6]. Despite its numerous properties, some drawbacks of HAp such as slow growth rate and the predilection of facilitating bacterial adherence and proliferation have been identified [7].

Due to their biodegradability, good biocompatibility, and great mechanical properties, various metals such as iron, zinc, magnesium, calcium, manganese, sodium, potassium, etc. have been viewed as ideal candidates for biomedical applications [8,9,10,11,12]. Amongst these metals, zinc has attracted considerable attention recently, because it exhibits great biocompatibility [13], osteoconductive properties [14], and has been reported to have a direct effect on the in vitro bone mineralization [15,16]. Studies have shown that comparative with magnesium, the degradation of zinc by fast corrosion does not form hydrogen gas pockets, which can inhibit the bone repair, forming calluses, and cortical defects [17]. Moreover, zinc exhibits a good in vivo degradation without creating corrosion byproducts, which are afterwards hard to eliminate by the human organism [18]. Zinc is also an essential trace element and is part of the human body [19,20], animals [21], and plants [22], participating in most of the principal basic biological functions. Zinc plays important roles in the metabolism of RNA and DNA, nucleic acid metabolism, apoptosis regulation, signal transduction, and gene expression [20]. Another important role of this metal is the direct activation of the aminoacyl-tRNA synthetase of osteoblastic cells, thus stimulating the cellular protein synthesis, and the inhibition of osteoclast-like cell formation of the marrow cells, which lead to osteoclastic bone resorption [23]. Due to these spectacular properties, and to the fact that zinc is able to promote the proliferation and differentiation of osteoblast cells, leading to an enhanced osteogenesis, the use of zinc in the development of materials designed to be employed as coating for implantable devices have attracted considerable attention in the last years [7,24]. However, studies have confirmed that hydroxyapatite coatings do not exhibit antimicrobial effects properties and in several cases contribute to the development of microbial cells. Taking into consideration this aspect, the use of metallic ions that possess antimicrobial properties embedded in the hydroxyapatite matrix has been proposed in the literature [7,25,26,27,28]. It has been reported the HAp properties could be greatly improved with the aid of different cationic and anionic substitutions, due to the fact that HAp has the ability to incorporate various substitute ions (Mg^2+^, Ag^+^, Ce^3+^, Eu^3+^, Sm^3+^, Cu^2+^, Mn^2+^, Zn^2+^, Na^+^, Sr^2+^, HPO_4_^2−^ or CO_3_^2−^) within its lattice [25,26,27,28]. Zinc is one of the most common elements involved in numerous functions in the human body. Due to the fact that zinc ions are found mostly in the human bone and hard tissues, they have the ability to facilitate the osteoblast proliferation and enhance the biomineralization and the bone formation. Considering all these aspects, composites based on zinc-doped hydroxyapatite could be suitable for being used in biomedical applications [7,25,26,27,28].

Furthermore, the development of zinc substituted hydroxyapatite (ZnHAp) powders has been given a considerable attention in the last decade due to the fact that both Zn and HAp are essential elements of the human hard tissue and separately have been reported to have major contribution in enhancing tissue and bone regeneration [29]. Recent papers have revealed that zinc ions possess antimicrobial activity, another particular and impressive property [7,30,31]. Stanic et al. [32] in their studies reported obtaining zinc-doped hydroxyapatite nanocrystals and proved their antimicrobial activity using two of the most common bacterial strains and a fungal strain. Studies performed by Thian et al. [33] have revealed that zinc-doped hydroxyapatite exhibits antibacterial properties. Even though the ZnHAp powders have been reported to exhibit antimicrobial properties, these properties do not maintain their level when ZnHAp coatings are involved. In their paper regarding the “in vitro antibacterial evaluation of sol-gel derived Zn^−^, Ag^−^, and (Zn + Ag) doped hydroxyapatite coatings against methicillin-resistant Staphylococcus aureus”, Samani et al. [34] reported the inhibition of the methicillin resistant *Staphylococcus aureus* (MRSA) by ZnHAp coatings.

Therefore, the development of zinc-doped hydroxyapatite is a current ongoing research topic and new approaches are thus required to prepare zinc-doped hydroxyapatite to achieve the optimal combination of both antibacterial efficacy and biocompatibility.

The aim of this study was to characterize the stability of undiluted bioceramic gels (7ZnHAp with x_Zn_ = 0, x_Zn_ = 0.07) by a non-destructive ultrasonic method. The HAp and 7ZnHAp samples were characterized by X-ray Diffraction (XRD), and also X-ray photoelectron spectroscopy (XPS). Moreover, ultrasound measurements have been used to characterize the HAp and 7ZnHAp samples.

## 2. Results and Discussions

In this study were presented the results obtained from the physico-chemical and biological characterization of bioceramic nano-powders (hydroxyapatite (HAp), x_Zn_ = 0 and zinc-doped hydroxyapatite (7ZnHAp), x_Zn_ = 0.07) obtained by sol-gel route [35].

**Figure 1:**
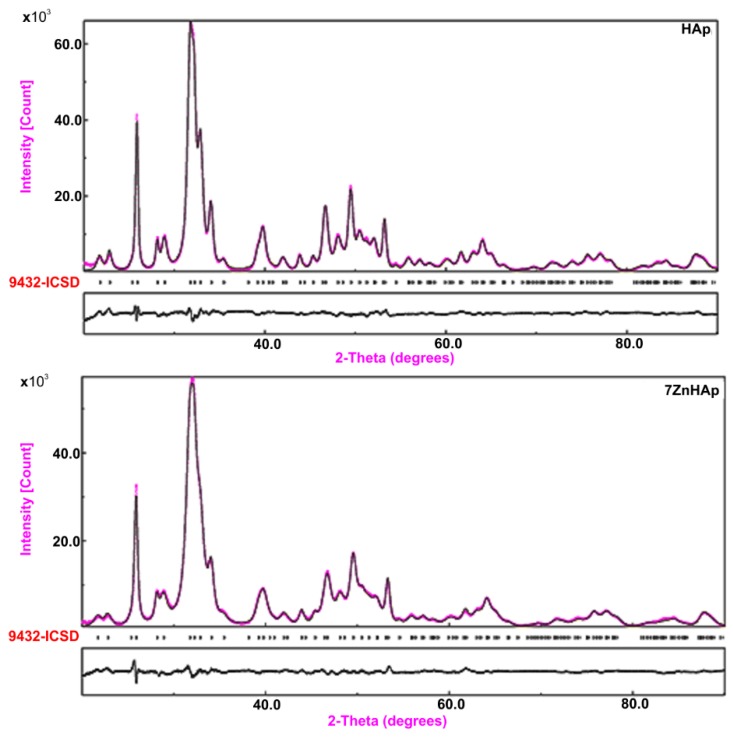
Rietveld processing of HAp and 7ZnHAp samples prepared by sol-gel method. Experimental data was depicted in pink while calculated data was depicted in a black line. Vertical lines represent the positions of diffraction lines of hexagonal hydroxyapatite (ICDD-PDF No. 9-432). The black line at the bottom of the figure represents the profile of the difference between experimental data and those calculated using the Rietveld method.

The XRD analysis of HAp and 7ZnHAp samples using the Rietveld refinement method revealed a single phase corresponding to the hexagonal hydroxyapatite. The comparison between the experimental and the calculated data obtained from the Rietveld refinement of the samples was presented in Figure 1. The experimental data are marked in blue and the calculated data are represented by a gray line. Vertical lines represent the positions of diffraction lines of hexagonal hydroxyapatite (ICDD-PDF No. 9-432). The gray line at the bottom of the figure represents the profile of the difference between experimental data and those calculated using the Rietveld method. The Rietveld refinement were realized using MAUD (Material Analysis Using Diffraction) program [36].

The processing of XRD data collected in the 2θ, 20–90° using the Rietveld method confirmed the formation of the monophasic hydroxyapatite structure belonging to the P6_3/m_ spatial group in all analyzed HAp and 7ZnHAp. The correctness of the Rietveld refinement of the analyzed samples was monitored by a number of parameters such as the index of the weighted profile R_wp_ and the index Χ. The Χ index is given by the ratio of R_wp_ and R_exp_ factors statistically estimated and represents the “correctness of the overlap between experimental and calculated data”. On the other hand, the R_Bragg_ factor determined using the Rietveld method is very useful because its value depends on the fit of the structural parameters. The values obtained for the final factors (R_wp_, R_exp_, and R_Bragg_) [37] for the HAp and 7ZnHAp samples using the Rietveld method are presented in Table 1. The theoretical values of the factors 𝑅 obtained for the HAp and 7ZnHAp samples are consistent with Toby’s theory [37]. Furthermore, complex diffraction spectra processing has demonstrated that the materials prepared by the sol-gel method exhibit the characteristics of the hexagonal hydroxyapatite with a good crystallinity. The peaks become slightly broad when the zinc concentration increased from x_Zn_ = 0 to x_Zn_ = 0.07. This behavior was caused by the small size effect of crystalline. Further, another supplementary phase was not identified.

**Figure 2:**
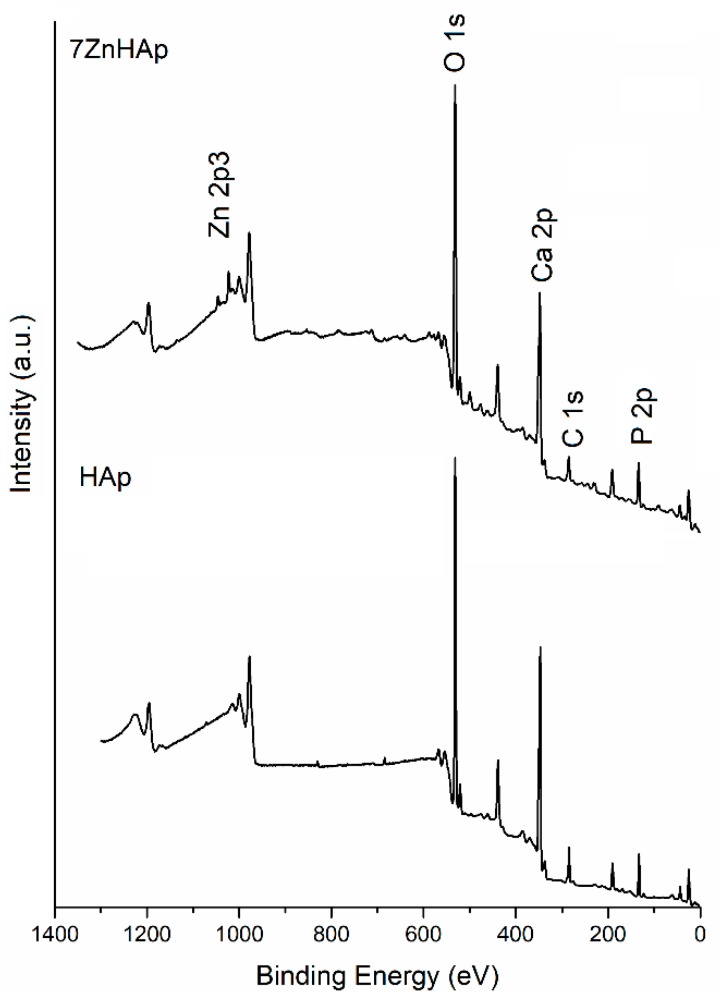
General XPS spectra of HAp and 7HApZn powders.

Information regarding the elemental composition of HAp and 7ZnHAp samples were obtained by the XPS analysis. XPS measurements revealed the presence of Zn^2+^ ions in the hydroxyapatite structure as a result of Ca^2+^ ions substitution during the synthesis process (7ZnHAp sample). The general spectra of the HAp and 7ZnHAp samples were shown in Figure 2.

**Figure 3:**
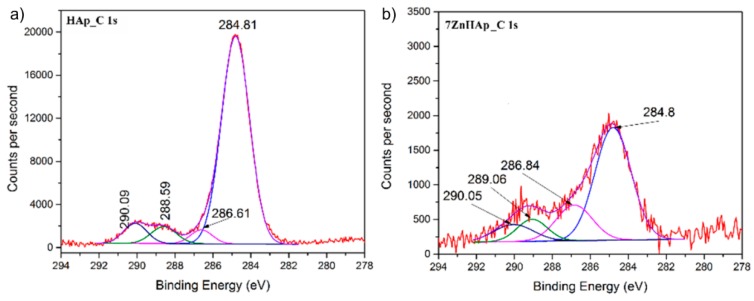
The high-resolution XPS spectra for C 1s of HAp (**a**) and 7ZnHAp (**b**) samples.

Peaks corresponding to C 1s, Ca 2p, P 2p, O 1s, and Zn 2p have been recorded for both samples investigated (x_Zn_ = 0 and x_Zn_ = 0.07). As expected in the XPS spectrum of HAp (x_Zn_ = 0) sample, the associated Zn 2p peak was not present. Moreover, in the course of this study, the high-resolution spectra obtained for C 1s, Ca 2p, P 2p, O 1s, and Zn 2p are presented and analyzed. The peak corresponding to C 1s was considered to have a binding energy value equal to 284.8 eV [38]. Figure 3 shows the high-resolution XPS spectra for C 1s and peaks of HAp and 7ZnHAp. It can be noticed that the value of the binding energy for the C 1s maxima remains unchanged after the substitution of the Ca^2+^ ions with the Zn^2+^ ions. A decomposition of the maxima of the corresponding energy interval C1s was performed for both samples. Peaks obtained after decomposition of C 1s were observed at the same binding energy for both samples (x_Zn_ = 0 and x_Zn_ = 0.07) irrespective of the zinc concentration. In the opinion of J. Serra et al. [39] the maximum positioned at 284.8 eV corresponds to C-C linkages or C-H bonds. Boyd et al. [40] identified the peak at 284.8 eV as being associated with C-C linkages and used it as a reference. The occurrence of the peak around 286 eV is associated with C-O-C linkages. This peak cannot be considered as a reference since its occurrence may also be due to traces of elements present in the analysis enclosure. The maximum at about 288.5 eV is attributed to C=O bonds while the peak around 290 eV could be associated with C-metal or O-C=O [41,42] linkages. Contamination with C was higher for HAp (x_Zn_ = 0) sample as this sample was measured after a longer period of exposure to the atmosphere. The sample with x_Zn_ = 0.07 was measured immediately after it was obtained. On the other hand, it was observed that the maxima associated with the binding energies widen and decrease in intensity in the 7ZnHAp sample.

**Figure 4:**
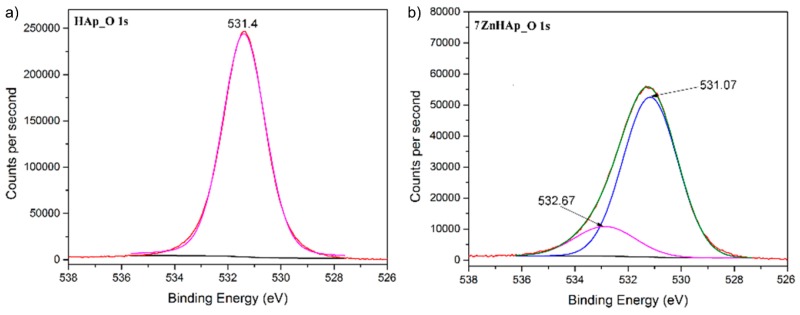
The high-resolution XPS spectra for O 1s of HAp (**a**) and 7ZnHAp (**b**) samples.

Figure 4 shows high-resolution XPS spectra for O 1s oxygen of HAp and 7ZnHAp samples. The peak associated to O 1s was located around 531.4 eV. Binding energies ranging of 531 to 531.5 eV could be ascribed to phosphates (PO_4_^−^), as it is widely reported in the literature. Following the deconvolution of the O 1s peak, in the case of HAp only one maximum was observed at a binding energy (EB) equal to 531.4 eV. For sample 7ZnHAp the associated O 1s peak was deconvolved in two peaks. The main peak was identified at 531.07 eV while the second peak was identified at a binding energy equal to 532.67 eV. The results of these studies are consistent with those previously reported by J.F. Moulder et al. [43]. Furthermore, G. Gaggiotti et al. [44] has shown that these peaks could be associated with surface hydroxyl (OH^−^) ions. On the other hand, T. Kawabe and co-workers [45] consider that the two oxygen species, for example O^−^ and OH^−^, can be associated with these peaks. Other previous studies have shown that the peak associated with the binding energy (EB) with values ranging from 531.0 to 531.5 eV [46,47,48] can be attributed to the O-adsorbed oxygen.

**Figure 5:**
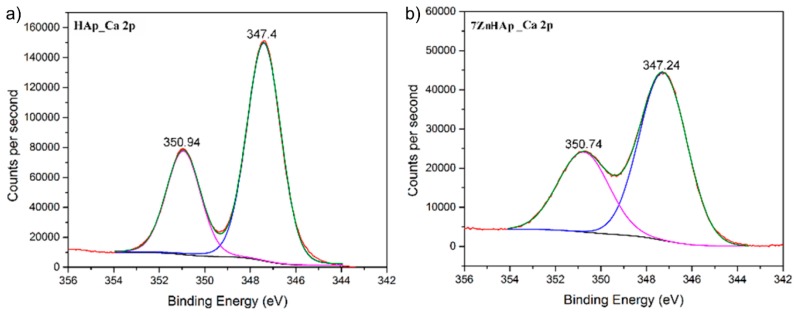
The high-resolution XPS spectra for Ca 2p of HAp (**a**) and 7HApZn samples (**b**).

The high-resolution XPS spectra of Ca 2p for the HAp and 7ZnHAp samples are shown in Figure 5. The high-resolution XPS spectra of Ca 2p for the HAp and 7ZnHAp samples have two peak values associated with the bonding energy values of 350.94 eV (Ca 2p^1/2^) and 347.27 eV (Ca 2p^3/2^), respectively. For the 7ZnHAp sample it was observed that the two peaks position are slightly shifted to lower values. The peak identified around 347.27 eV that was associated with Ca 2p^3/2^ shows that calcium atoms are bound to phosphate groups (PO_4_^3−^). Moving this peak to lower values for the 7ZnHAp sample could indicate a decrease in the crystallinity of this sample [49].

**Figure 6:**
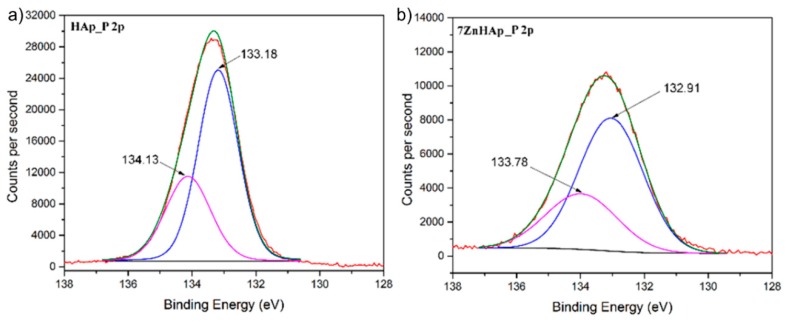
The high-resolution XPS spectra for P 2p of Hap (**a**) and 7HApZn samples (**b**).

High-resolution spectrum associated of P 2p peak and its deconvolution for HAp samples (x_Zn_ = 0) and 7ZnHAp (x_Zn_ = 0.07) are shown in Figure 6. The XPS spectrum of P 2p consists of two lines, P 2p^3/2^ and P 2p^1/2^ spaced at around 0.9eV one from the other. T. F. Stoica et al. [50] showed that the peak associated to P 2p was located at 133.4 eV. Moreover, in previously reported studies, C. Battistoni et al. [51] has shown that the peaks in the photoelectron spectra for Ca and P are characteristic of the oxidation states (Ca^2+^ and P^5+^) characteristic of hydroxyapatite. The binding energies associated with the two peaks resulting from the deconvolution decrease for the 7ZnHap sample. It can be observed that for the HAp sample (x_Zn_ = 0) the two peaks are associated with the binding energies around the values of 134.13 eV and 133.18 eV, while for the sample 7ZnHAp (x_Zn_ = 0.07) the energies corresponding to the two peaks are 133.78 eV and 132.91 eV.

**Figure 7:**
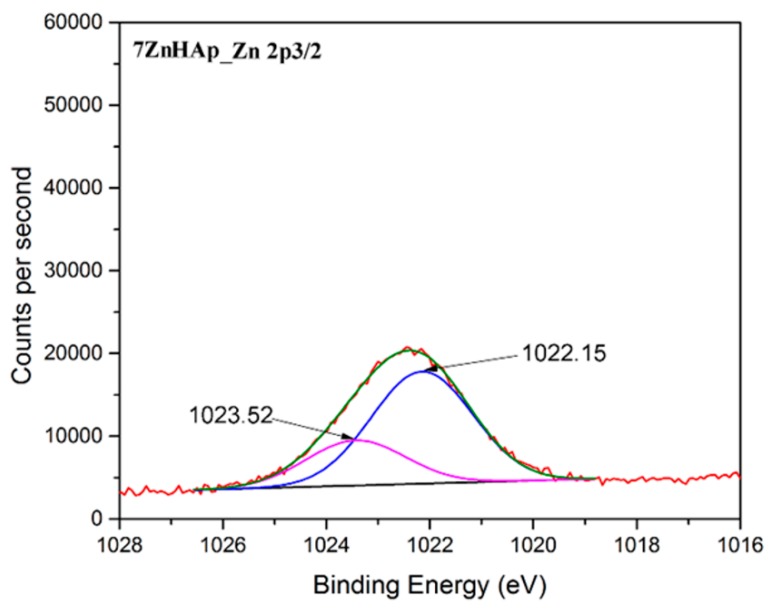
The high-resolution XPS spectrum for Zn 2p of and 7HApZnsamples.

As expected in the XPS spectrum of 7ZnHAp the associated Zn 2p^3/2^ peak was present (Figure 7). It can be noticed that the value of the binding energy for the Zn 2p^3/2^ maxima peak was observed around the binding energy of approximately 1022.4 eV [52] in good accordance to data reported in the literature [53]. This suggests that the valence of zinc did not change following the substitution of the Ca^2+^ ions with Zn^2+^ [54].

Supplementary information about HAp and 7ZnHAp gels used in medical applications were achieved the first time by ultrasound measurements. This technique has allowed us to evaluate the stability of gels obtained in concentrated form. The stability of resulted gels was evaluated after being stirred for 15 min at room temperature. To solve the problems of interaction between the ultrasonic elastic waves propagating in the analyzed gels and the solid nanoparticles that form these gels, the Finite Elements Method (FEM) was be used to simulate the wave propagation. For a 0.1% accuracy in velocity computations, a fluid domain of 100 × 100 × 2000 nm was considered, in which the nanoparticles are randomly placed. For a good accuracy of the results, sufficient space, for more than 5 nanoparticles along the small side of the domain, was provided for a random positioning. The random insertion of nanoparticles with a given volumetric ratio in a parallelepiped domain, was generated by an original algorithm implemented in the MATLAB program. [55].

A number of 153 spheres without touching or intersections between spheres were thus inserted. Moreover, a possible random variation (of 10% in this case) of the nanoparticle radius was implemented, in agreement with the actual dispersion of the nanoparticles. The geometry thus obtained for elastic solids (the 153 spheres) and the surrounding water was transferred to the FEM model implemented in COMSOL [56], using its dedicated fluid-solid acoustic interaction module. The continuity conditions between the two domains were established by the continuity equation for displacements (or acceleration) and the continuity of the pressure in the fluid transferred as mechanical stress to the solid particles. At one end of the parallelepiped section, a time harmonic variable pressure P, with a frequency of f = 5 MHz has been applied. At the opposite end, the “free radiation condition” for a flat wave was chosen. The most realistic choice is to impose the periodicity of the boundary conditions between the opposite rectangular surfaces of the parallelepiped. This choice, along with the random distribution of the spheres, best corresponds to the infinite theoretical field in the lateral direction (perpendicular to the propagation direction). The mesh of 250,000 tetrahedral finite elements covers the domain with enough detail, to describe correctly the shape of the spheres. The solution is obtained for five frequencies centered around 5 MHz (between 3.75 MHz and 6.25 MHz).

For a given value of the temperature, the plane wave in pure water propagates at a speed *c*_0_. The distance dependency of the pressure value (along the FEM model of length *L*) is given by:

(1) $$p_{a}=P\cos\left(\omega L/c_{0}\right)$$

The inverse procedure was applied for nanoparticles in suspension. The pressure *p_a_* is determined from the calculated FEM solution for the opposite wall (*z* = *L*), resulting the equivalent speed in the biphasic environment:

(2) $$c=\left(\omega L\right)/\cos^{-1}\left(p_{a}/P\right)$$

**Figure 8:**
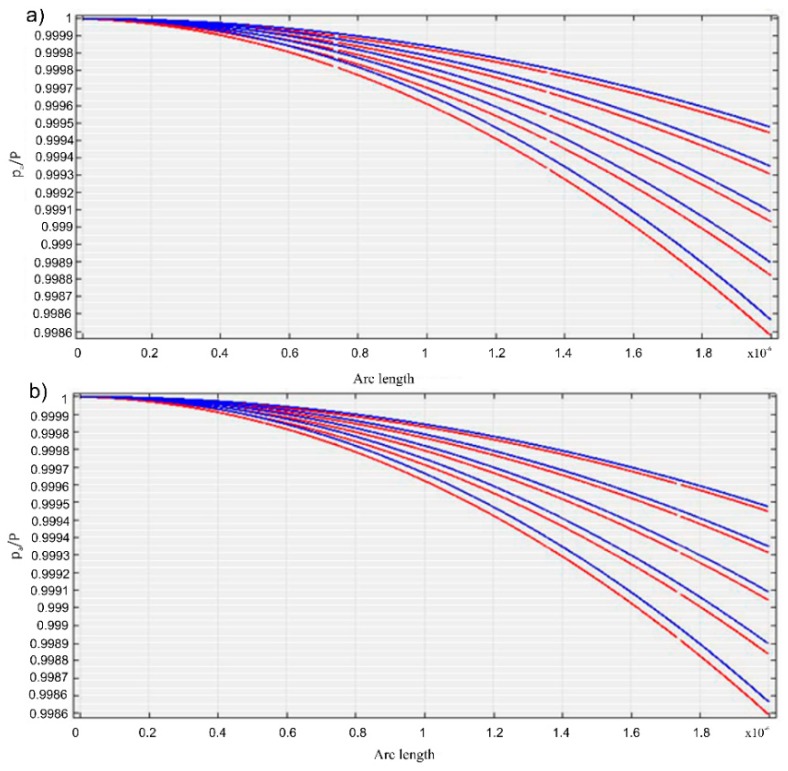
Acoustic pressure in the biphasic environment for HAp (red) for five frequencies and in water (blue) (**a**); Acoustic pressure in the biphasic environment for 7ZnHAp (red) for five frequencies and in water (blue) (**b**).

**Figure 9:**
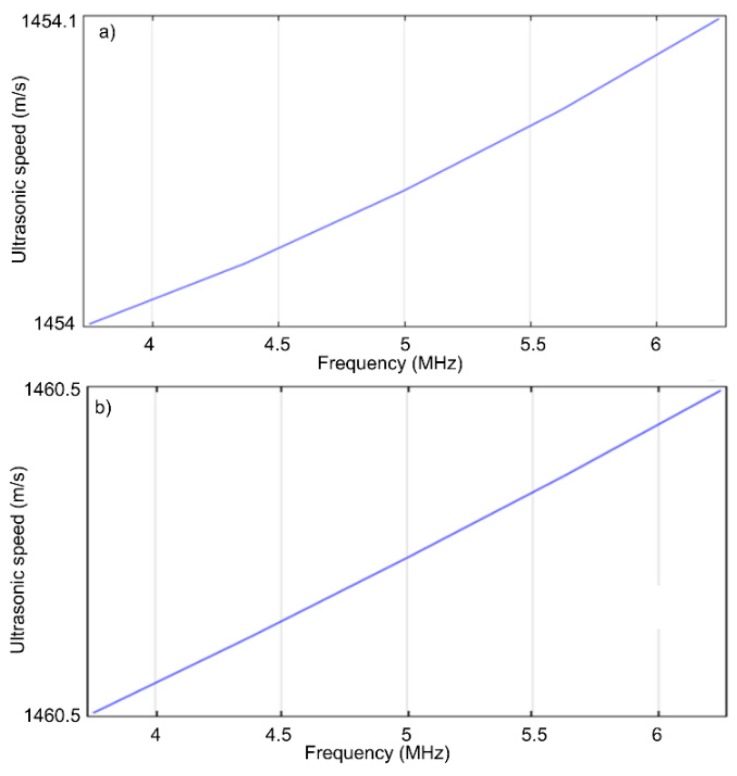
Speed in the biphasic environment for the frequency interval studied for HAp (**a**) and 7ZnHAp (**b**).

The *p_a_/P* ratios along the axis, represented in red for the five frequencies (Figure 8), were obtained for the HAp (R = 13 nm, density ρs = 1.0129 kg/m^3^ and a 5% volumetric concentration) and 7ZnHAp (R = 9.7 nm, density ρs = 1.0062 kg/m^3^ and a volumetric concentration of 5%) samples. The rate in the biphasic medium for the HAp and ZnHAp samples in the frequency range studied was shown in Figure 9.

The velocity values in the biphasic media according to frequency for HAp (solid phase: E = 10 Gpa, ν = 0.3, 5% water volume ratio, R = 13nm) and 7ZnHAp (solid phase: E = 6 Gpa, ν = 0.3, volumetric ratio in water 5%, R = 9.7 nm) dispersions were presented in Table 2. The ultrasonic velocity obtained for the values E = 10 GPa and ν = 0.3 has an almost identical value to that resulting from the experimental data obtained for the dispersion of HAp 1454.17 ± 1.06 m/s) and ZnHAp (1476.02 ± 0.15 m/s).

**Figure 10:**
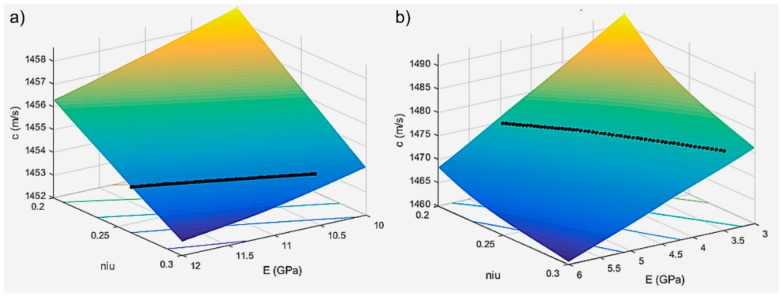
Ultrasonic velocity through the HAp (**a**) and 7ZnHAp (**b**) dispersions according to Young (E) module values. The black lines indicate the interpolated values.

**Figure 11:**
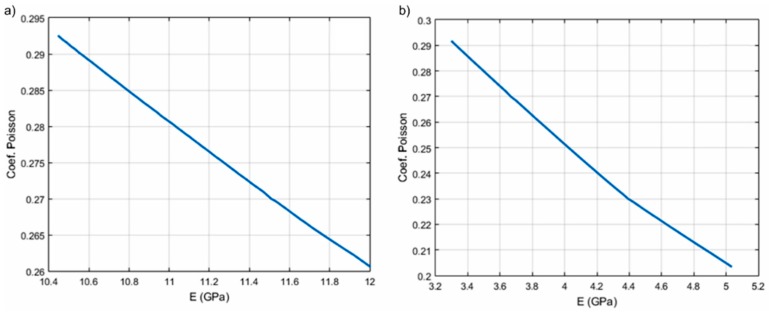
The dependence of Poisson coefficients (ν) and the Young (E) modulus for HAp (**a**) and 7ZnHAp (**b**).

Using an iterative procedure, the speed in the biphasic medium, providing information about the elastic properties of the solid environment with a minimum error in a two-variable domain was determined by interpolation (E, ν). Based on the results obtained from modeling for a series of values of the Young modulus and the Poisson coefficient, an interpolation program *c* (E, ν) was performed. Ultrasound velocity values through the HAp and 7ZnHAp dispersions were determined for different values of the parameter pair (E, ν). The results obtained for the ultrasound velocity according to the pairs of parameters E and ν by the studied dispersions were presented in Table 3. The results obtained after the algorithm for the interpolation has been applied of the velocity *c* versus (E, ν) were presented in Figure 10 and Figure 11.

A major inconvenience of this method is represented by the small size of the nanoparticles (the nanoparticle’s radius is ~10 nm) compared to the wavelength of the ultrasonic waves which propagate through water (~0.3 mm at 5 MHz). In a volume of 100 × 100 × 300,000 nm with a volumetric concentration of 5%, there are about 30,000 nanoparticles, which is obviously a problem that cannot be solved. The main difficulty in applying this FEM model is represented by the four rectangular lateral surfaces. The incident pressure propagating in the fluid acts on each elastic sphere and these spheres are also acted upon by multiple pressure scattering produced by the rest of the spheres. For this reason, through the side surfaces of the model, the laterally scattered pressure waves, produced by the vibrating spheres, will be affected by non-realistic reflections at the rigid walls, which behave as a waveguide. In this context, considering the rigid wall as boundary conditions are not appropriate. For this reason, our model uses periodic boundary conditions on the lateral sides, so that in the total pressure is included the pressure coming from the spheres in the surrounding identical parallelepipedic domains, by periodicity.

Using a model based on finite elements in COMSOL and through specialized algorithms in MATLAB, a preliminary study was conducted on the influence of ultrasonic waves on HAp and 7ZnHAp gels. Ultrasonic velocity was also determined from this model for a suspension of elastic nanoparticles, in which E (Young’s mode) and ν (Poisson coefficient) are main parameters. The values of the ultrasonic velocity determined for different pairs of parameters (E, ν) were validated experimentally. It can be concluded that the model used is suitable for the characterization of hydroxyapatite-based ceramics.

The biphasic velocity values obtained for different frequencies for the HAp and 7ZnHAp dispersions confirmed the stability of the solutions. Moreover, the ultrasound velocity values determined for different values of the parameter pair (E, ν), established that the 7ZnHAp dispersions were much more stable (Table 3). The parameter pair (E, ν) increases with the stability of solutions. As can be seen, 7ZnHAp was more stable. The increase in stability could have been influenced by the lower particle size in the 7ZnHAp sample.

The evaluation of gel stability by ultrasound measurements allowed us to assess the stability of undiluted gels by a non-destructive method. It is important to underline that information on gel stability by ultrasound measurements is more accurate than that obtained by traditional methods such as ζ-potential method (where the solutions analyzed are diluted). On the other hand, this non-destructive gel analysis method is an important step in studying gels especially when they are well structured.

## 3. Materials and Methods

The precursors used in our study were calcium nitrate tetrahydrate (Ca(NO_3_)_2_·4H_2_O as a source of calcium ions and diammonium phosphate (NH_4_)_2_HPO_4_) as the phosphorus precursors and zinc nitrate hexahydrate (Zn(NO_3_)_6_·6H_2_O). The ammonium hydroxide (NH_4_OH), ethanol, and deionized water were also utilized. Zn-doped hydroxyapatite Ca_10−x_Zn_x_(PO_4_)_6_(OH)_2_, (ZnHAp), with various Zn/(Zn + Ca) ion ratios (x_Zn_ = 0 and x_Zn_ = 0.07) was obtained using an adapted sol-gel processing route [57]. The molar ratio of precursors, [Ca + Zn]/P, in the ZnHAp sol was minded to be equal to 1.67 according to our previous studies [58,59]. (NH_4_)_2_HPO_4_ and Zn(NO_3_)_6_·6H_2_O were dissolved in absolute ethanol under continuous stirring. Ca(NO_3_)_2_·4H_2_O was also dissolved in absolute ethanol. The solution based on phosphate and zinc was slowly added into the solution based on calcium. To keep the pH constant at 11, NH_4_OH was added. In this experiment, the resulting solution was stirred continuously for 48 h at 100 °C until the formation of a gel. The resulting gels were washed five times with double deionized water and ethanol. After washing, the gels were dispersed in deionized water under continuous stirring for 6 h. The final gels thus obtained were analyzed by various techniques.

X-ray diffraction measurements were performed using a Bruker D8 (Bruker, Karlsruhe, Germany) advanced diffractometer that uses a CuKα (λ = 1.5418 Å) radiation with a nickel filter. The diffractometer is equipped with a high-resolution, one-dimensional detector Lynx Eye type (Bruker, Karlsruhe, Germany). The measurements were made in the range of 2θ, 20°–90°, with a step of 0.02°, using an acquisition time of 34 s/step. For a complex DRX analysis of the nanopowders studied, the diffractograms were processed using the Material Analysis Using Diffraction (MAUD) (L. Lutteroti, University of Trento, Italy) program (http://maud.radiographema.eu/) [60] using the Rietveld refining and “POPA rules”. Following this detailed analysis, we could determine the structure, network parameters, and the average size of crystals.

X-ray photoelectron spectroscopy measurements (XPS) were performed with a Multimethod SPECS surface analysis system. The device operates with Al K_α_ (1486.6 eV) monochromatic radiation. The vacuum in the analysis chamber was *p* ~3 × 10^−9^ torr. X-radiation is emitted by an Al-cathode, the voltage being U = 12.5 kV and the filament emission current I = 20 m. A FG40 cannon that produces an electron beam with energy of 2 eV and a power of 0.3 mA was used to compensate for the load. For acquisition of XPS spectra, an energy window w = 20 eV with resolution R = 20 eV and 400 recording channels was used. For Zn doped samples, the monochromatic X-ray source (Specs XR-50 M) was used in the FOCUS mode in order to obtain an X-radiation with a FWHM (Full Width at Half maximum) less than 0.25 eV. This has been done to increase the resolution of XPS spectra of Zn-doped samples, to have the ability to observe small chemical shifts. But indeed, the X-Rays flux is reduced in the FOCUS mode. The obtained XPS spectra were analyzed and processed using Spectral Data Processor v2.3 (SDP) [23].

**Figure 12:**
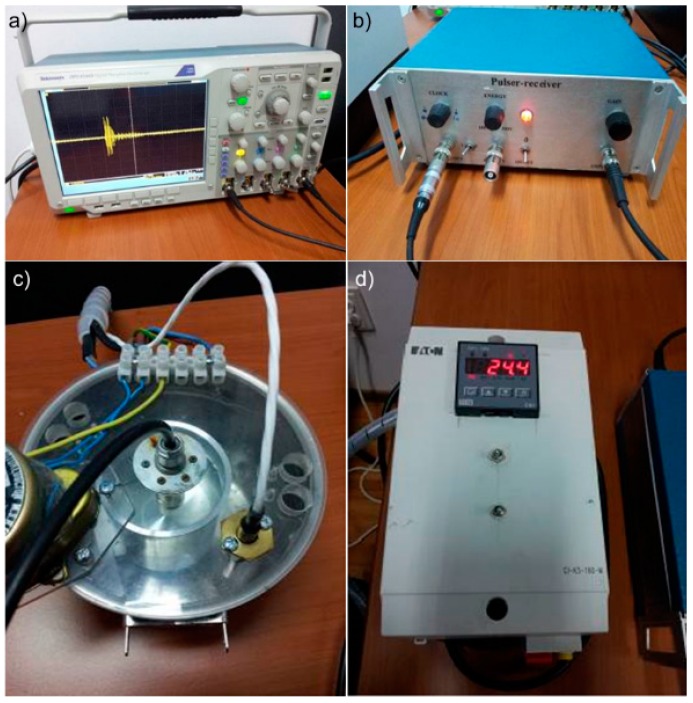
Experimental setup: Oscilloscope used to display and store the measured signals (**a**); pulser-receiver (**b**); thermo-controlled container (**c**) and the electronic control module (**d**).

The ultrasound characterization of HAp and ZnHAp suspensions were mixed continuously on a magnetic stirrer for 30 min. Then the suspensions were placed in a thermally controlled container. The resulting signals were displayed and stored on an oscilloscope (Tektronix, Inc., Beaverton, OR, USA). Instruments, oscilloscope (Tektronix, Inc., Beaverton, OR, USA) and Pulser-receiver, JSR Ultrasonics DPR300 (Imaginant Inc., Pittsford, NY, USA), used for the ultrasonographic characterization, of hydroxyapatite dispersions and zinc-doped hydroxyapatite are shown in Figure 12.

## 4. Conclusions

In the current study the hydroxyapatite and zinc-doped hydroxyapatite were successfully synthesized by sol-gel route at room temperature using Ca(NO_3_)_2_·4H_2_O, (NH_4_)_2_HPO_4_ and Zn(NO_3_)_2_·6H_2_O as calcium, phosphorus precursors, and zinc precursors. The phase composition and elemental composition of HAp and 7ZnHAp samples were obtained by XRD and XPS analysis. Rietveld analysis revealed the HAp and 7ZnHAp powders with monophasic hydroxyapatite structure belonging to the P6_3/m_ spatial group were obtained. The presence of constituent elements in the analyzed samples were achieved by XPS analysis. The information about the stability of HAp and 7ZnHAp gel samples were obtained from ultrasound measurements using the Finite Elements Method (FEM) to simulate the wave propagation. The model we have proposed in this study was first used in gel analysis. Our model proposed to use the periodic boundary conditions on the lateral sides, so that in the total pressure is included the pressure coming from the spheres in the surrounding identical parallelepipedic domains. Our study demonstrated that the non-destructive ultrasonic method is suitable to characterize undiluted bioceramic gels.

## Figures and Tables

**Table 1:** Values obtained for R final factors (R_wp_, R_exp_ şi R_Bragg_).

Sample	R_wp_ (%)	R_exp_ (%)	R_Bragg_ (%)
HAp	2.4121	0.9341	1.8474
7ZnHAp	2.3299	0.9188	1.7531

**Table 2:** The velocity values in biphasic medium versus frequencies for HAp and 7ZnHAp dispersions (solid phase: E = 2 Gpa, ν = 0.3, volumetric ratio in water 5%, R = 4.98 nm).

	HAp	7ZnHAp
F (Hz)	velocity (m/s)	velocity (m/s)
3.7500 × 10^6^	1454.0	1460.5
4.3750 × 10^6^	1454.1	1460.5
5.0000 × 10^6^	1454.1	1460.5
5.6250 × 10^6^	1454.1	1460.5
6.2500 × 10^6^	1454.1	1460.5

**Table 3:** The speeds in the biphasic medium according to E and ν parameters for the studied dispersions.

HAp				7ZnHAp			
E/ν	Ν = 0.2	Ν = 0.25	Ν = 0.3	E/ν	Ν = 0.2	Ν = 0.25	Ν = 0.3
E = 10	1458.6	1456.3	1454.1	E = 3	1492.5	1484.3	1476.2
E = 11	1457.4	1455.3	1453.2	E = 4	1480.2	1468.3	1468.3
E = 12	1456.3	1454.4	1452.6	E = 6	1468.3	1464.4	1460.5
